# Supplementary material for: Inflammation and Syndecan-4 Shedding from Cardiac Cells in Ischemic and Non-Ischemic Heart Disease
Source: Biomedicines. 2023 Apr 1;11(4):1066. doi: 10.3390/biomedicines11041066 (PMC10135943; doi:10.3390/biomedicines11041066)
Supplement: Supplementary file 1 [file biomedicines-11-01066-s001.zip › Supplementary Material 2nd proof.pdf]

Supplementary Material

Table S1. Corresponding data to Figure 1C

Serum Syndecan-4 levels (ng/mL)

Radial artery	Coronary sinus
34,43	48,92
28,02	50,76
1,6	3,66
1,7	6,69
1,14	14,8
2,46	5,05
5,04	8,79
7,76	1,09
5,58	10,13
2,14	0,72
0,18	0,3
1,77	5,61
2,12	2,18
2,76	0,78
0,83	1,43

Table S2. Corresponding data to Figure 3

Serum Syndecan-4 levels (ng/mL)

Non-inflammatory DCM	Inflammatory DCM	Myocarditis	Pericarditis	Perimyocarditis	Day 0 post-MI	Day 3 post-MI	Day 30 post-MI	Healthy controls
19,56	9,9	7,92	12,98	22,25	14,82	19,95	17,58	21,092
16,54	9,4	12,78	13,98	19,39	15,93	21,66	11	9,865
32,25	24,1	23,86	13,67	12,54	12,68	20,2	18,6	14,733
15,46	13,5	11,76		14,88	22,1	23,95	22,11	12,923
23,81	12,8	2,44		10,6	13,93	7,614	14,77	7,8745
16,29	14,03	3,04		15,22	19,59	21,87	18,2	
28,58	9,7	13,9		24,15	14,23	11,88	16,77	
22,19	11,97	10,37		18,95	10,4	10,83	12,24	
20,05	26,03	24,33		16,19	16,9	13,7	24,3	
38,72	14,01	11,35		11,91	8,3	11,4	14,8	
13,78	22,73	8,44		13,1	19,2	14,8	19,3	
22,8	24,4	31,28		36,99	16,6	19,1	19,2	
25,69	16,95	17,33		37,05	12,8	13,4	11,4	
16,74	14,97	14,78		10,14	15,8	15,5	15,1	
15,89	8,2	11,29		12,91	9,4	12,1	10	
25,41	8,28			14,04	14,7	15,9	16,6	

11,37	29,92			15,49	32,2	26	20,5	
24,39	10,95			17,12	13,5	13,3	12,6	
17,27	16,04			16,85	11,2	8,9	13,1	
15,48	12,87			20,81	24,7	18,2	24	
19,67	10,06			13,02	14,4	13	12,9	
10,11	13,73			4,79	23,1	19,8	19,4	
24,05	9,77			23,44	11,6	11,7	13,6	
14,51	23,27				9,5	13,5	10,3	
18,59	6,95				15,2	12,65	13,87	
8,42	12,69				9,566	9,153	9	
14,68	18,31				24,08	24,21	21,06	
14,77	20,98				15,32	15,67	13,08	
8,03	16,72				3,759	14,99	15,91	
17,13	11,19				26,64	32,24	34,8	
21,37	17,45				26,95	26,38	20,54	
9,32	11,51				9,954	16,17	15,03	
10,36	11,57				19,39	17,88	18,08	
12,76	23,8				19,32	20,07	21,03	
12,92	16,12				14,43	15,9	19,76	
13,86	15,43				11,78	8,324	8,329	
13,27	32,44				6,523	19,84	17,31	
14,43	22,22				16,65	16,59	17,72	
13,53	13,49				7,551	18,12	22,49	
15,43	8,82				12,01	20,81	15,96	
11,6	14,53				15,05	14,16	21,65	
26,55	16,76				17,52	19,38	17,22	
12,83	11,41				14,56	15,27	17,48	
12,9	13,28				22,57	22,22	22,25	
9,51	16,19				14,29	11,56	10,44	
5,32	23,93				12,81	13,47	11,67	
19,06	20,92				13	11,4	9,073	
13,63	23,69				19,38	15,14	8,43	
6,51	14,62				13,94	17,64	13,17	
10,93	30,83				1,218	9,613	9,853	
15,13	20,74				13,71	13,82	11,75	
11,81	11,71				10,53	12,78	9,899	
10,09	23,78				14,41	14,03	13,44	
14	15,46				15,73	13,01	10,7	
12,03	16,46				13,98	9,853	11,23	
11,48	12,72				2,711	15,32	14,42	
15,19	34,01				10,31	13,86	16,08	
23,86	19,78				10,76	10,39	7,632	

22,76	11,49				11,86	10,54	12,81	
25,7	10,83				21,59	21,73	13,88	
14,95	17,48				7,526	17,66	16,1	
22,85	11,51				14,09	13,26	11,44	
14,67	11,18				9,898	10,29	6,951	
32,39	13,4				4,969	7,176	5,944	
32,87	13,51				14,06	10,16	11,51	
17,49	10,66				13,24	10,48	13,08	
28,37	18,93				19,31	17,68	14,21	
10	21,61				17,49	15,62	17,24	
18,64	12,95				4,615	13,62	13,36	
15,62	15,81				6,854	21,39	20,57	
12,29	16,99				12,31	13,11	15,09	
21,88					17,83	18,82	18,38	
11,59					10,64	20,98	15,58	
16,55					5,779	16,02	14,49	
14,62					5,569	14,76	10,36	
13,28					3,799	14,48	11,49	
20,41					17,26	17,14	12,92	
12,63					4,656	11,93	13,41	
16,48					19,16	21,84	11,6	
12,94					9,009	8,607	8,341	
10,52					16,2	21,57	15,13	
18,43					2,51	5,844	5,987	
24,79					1,055	19,5	25,71	
10,77					14,13	15,92	14,77	
6,9					18,78	16,03	21,09	
11,58					15,04	14,26	6,292	
17,89					18,44	13,13	15,02	
14,52					21,86	17,1	20,83	
18,24					29,9	30,33	26,62	
18,73					12,4	13,49	11,7	
11,02					17,88	17,7	15,85	
8,85					11,4	13,36	12,5	
11,08					25,99	16,17	18,53	
10,83					10,91	9,74	10,74	
8,79					18,15	24,2	24,02	
10,99					29,31	21,68	17,39	
36,25					1,988	11,18	13,88	
5,63					3,996	16,65	24,45	
10,47					3,547	17,53	19,08	
25,68					16,18	14,65	13,06	

9,05					21,46	20,72	18,72	
11,15					3,51	24,2	16,49	
18,85					11,98	18,47	14,75	
22,25					18,08	16,78	14,97	
23,57					13,96	16,06	15,66	
12,79					12,98	14,93	13,54	
10,73					18,65	19,33	19,82	
10,6					27,52	21,83	24,77	
14,25					12,88	15,21	17,94	
21,82					11,87	13,75	12,58	
16,63					19,64	22,96	24,02	
9,2					15,31	18,83	20,37	
19,31					28,68	24,97	25,83	
14,74					0,856	11,42	8,491	
10,57					27,11	23,72	24,77	
8,07					19,81	23,98	16,48	
22,72					18,35	16,25	17,72	
10,92					23,25	23,6	27,8	
10,06					12,06	13,86	12,81	
13,87								
20,61								
8,58								
24,66								
13,05								
19,14								
16,55								
11,81								
9,4								
16,69								
15,56								
18,73								
10,69								
18,11								
9,12								
16,26								
8,45								
16,42								
19,96								
12,94								
19,42								
15,69								
13,61								

13,05								
14,23								
21,76								
16,26								
9,84								
10,85								
23,08								
16,88								
15,01								
30,08								
14,41								
11,12								
14,26								
9,3								
12,14								
17,86								
15,37								
16,73								
5,52								
12,99								
11,27								
15,37								
16,69								
27,49								
12,08								
8,44								
14,89								
16,91								
14,03								
8,47								
15,25								
17,11								
9,01								
14,51								
9,12								
17,44								
8,65								
25,4								
14,81								
12,61								
14,15								
21,35								

8,02								
23,29								
7,94								
13,84								
9,4								
11								
16,72								
13,07								
13,81								
18,9								
13								
15,6								
21,13								
9,25								
10,09								
12,25								
15,3								
17,62								
14,16								
11,66								
11,84								
5,97								
13,19								
4,36								
16,17								
17,99								
11,56								
18,45								
5,92								
9,57								
15,72								
8,25								
17,1								
9,37								
10,08								
24,28								
13,16								
30,37								
4,02								
10,82								
5,67								
14,9								

10,15								
12,35								
16,41								
9,56								
9,17								
13,77								
14,84								
13,74								
6,24								
13,56								
12,45								
14,88								
12,31								
14,74								
10,77								
11,93								
21,88								
11,92								
13,29								
13,11								
5,85								
18,97								
14,77								
11,2								
17,3								
14,54								
7,49								
3,27								
11,57								
13,14								
8,38								
8,64								
12,04								
8,03								
27,85								
12,4								
18,84								
9,84								
12,77								
9,03								
10,32								
10,96								

25,75								
7,22								
17,95								
18,43								
14,51								
19,94								
17,8								
15,42								
14,65								
27,42								
12,58								
11,58								
8,51								
22,19								
14,33								
10,9								
19,91								
14								
36,3								
21,07								
37,09								
8,63								
12,28								
8,18								
17,89								
11,48								
10,22								
23,92								
13,44								
19,55								
8,18								
14,24								
14,71								
8,6								
12,98								
28,64								
8,11								
28,57								
12,19								
19,36								
29,58								
13,31								

11,03								
26								
12,78								
12,81								
26,75								
21,04								
17								
11,58								

Table S3. Corresponding data to Figure 4A

Relative *Sdc4/Rpl4* mRNA

non-stimulated	IL-1Ra	IL-1β	IL-1β + IL-1Ra
1,1116	1,1567	2,3329	1,4519
0,9419	1,046	2,9565	1,5964
0,9465	0,9639	2,7619	1,3468
0,9302	1,1859	5,0069	2,0079
0,7654	0,9988	3,5485	1,7049
1,3043	0,9604	3,581	2,292

Table S4. Corresponding data to Figure 4B

Relative syndecan-4/vinculin protein

Full length (FL)				Cellular fragment (CF)			
non-stimulated	IL-1Ra	IL-1β	IL-1β + IL-1Ra	non-stimulated	IL-1Ra	IL-1β	IL-1β + IL-1Ra
1,458	0,973	1,294	0,908	0,723	0,567	1,006	1,032
0,765	0,429	1,485	1,179	1,515	0,528	1,291	1,229
0,777	0,424	0,795	0,834	0,762	0,498	1,329	0,919
1,021	0,152	3,222	2,671	0,684	0,285	2,827	2,957
0,605	0,13	2,209	2,372	1,33	0,371	2,517	2,401
1,374	0,124	3,093	3	0,986	0,288	3,107	1,996
0,652	0,53	6,695	4,504	0,668	0,581	2,116	2,203
1,008	0,896	7,804	3,688	0,823	0,992	3,709	2,908
1,341	0,918	7,992	3,381	1,508	1,265	4,248	3,067

Table S5. Corresponding data to Figure 4C

Relative *Sdc4/Rpl4* mRNA

non-stimulated	Infliximab	TNFα	TNFα + Infliximab
0,9281	0,5228	1,8688	0,3994
0,9527	0,9589	1,4833	2,0183
1,1191	1,123	1,8806	2,0758
0,9779	1,0405	2,2771	2,3273
1,0319	0,9898		2,4122
0,9902	1,1238	2,2708	2,3891

Table S6. Corresponding data to Figure 4D

Relative syndecan-4/vinculin protein

Full length (FL)				Cellular fragment (CF)			
non-stimulated	Infliximab	TNFα	TNFα + Infliximab	non-stimulated	Infliximab	TNFα	TNFα + Infliximab
1,213	1,784	3,392	1,783	1,145	2,184	5,121	3,589
0,462	0,697	2,842	2,975	0,511	0,668	4,267	6,305
1,325	2,249	3,75	3,574	1,344	1,586	5,064	6,145
0,883	0,882	1,074	0,912	1,357	0,751	1,175	1,007
1,031	0,633	1,035	1,41	0,835	0,345	0,672	1,054
1,086	0,696	1,13	1,735	0,808	0,445	0,813	1,209
0,857	0,654	5,247	2,748	1,137	0,69	3,404	2,315
1,039	0,502	4,354	3,015	0,719	0,614	3,685	3,818
1,104	0,78	3,274	3,489	1,145	1,165	4,022	2,998

Table S7. Corresponding data to Figure 5A

Relative *Sdc4/Rpl4* mRNA

non-stimulated	IL-1Ra	IL-1β	IL-1β + IL-1Ra
1,1534	0,953	7,9833	5,6319
1,0308	1,135	7,9877	5,2042
0,8158	0,7857	10,712	3,5004
0,9242	0,9054	6,0079	2,9542
1,0939	0,9345	7,3245	3,6307
0,9819	1,0052	6,4363	2,5985

Table S8. Corresponding data to Figure 5B

Relative syndecan-4/vinculin protein

Full length (FL)				Cellular fragment (CF)			
non-stimulated	IL-1Ra	IL-1β	IL-1β + IL-1Ra	non-stimulated	IL-1Ra	IL-1β	IL-1β + IL-1Ra
1,496	1,744	14,959	7,707	1,06	1,042	9,073	6,585
0,691	0,756	10,79	11,275	1,074	0,99	8,755	7,211
0,813	0,856	18,111	10,828	0,866	0,849	8,269	6,169
1,394	1,055	15,063	8,649	0,648	0,719	10,386	6,722
0,525	0,925	17,956	15,911	0,88	0,732	12,354	12,024
1,081	1,389	23,676	20,107	1,471	1,247	21,2	15,854
1,034	1,605	16,246	6,799	1,334	0,85	17,528	10,158
1,229	1,449	10,176	2,682	0,88	1,044	10,641	5,097
0,737	0,797	9,429	2,646	0,786	0,34	14,796	9,195
0,806	1,55	32,367	19,371	0,669	1,734	19,097	13,029
1,194	1,814	34,508	19,647	0,664	1,915	16,513	14,805
1	1,842	38,676	25,834	1,666	3,847	29,156	24,356

Table S9. Corresponding data to Figure 5C

Relative *Sdc4/Rpl4* mRNA

non-stimulated	Infliximab	TNFα	TNFα + Infliximab
0,961	1,1159	2,2681	1,7718
1,1833	1,0586	2,4382	2,0582
0,8557	0,9036	2,5953	2,2041
1,0011	0,9006	2,3093	2,1074
0,8749	0,8801	2,2897	2,1799
1,124	0,9728	2,3877	2,0301

Table S10. Corresponding data to Figure 5D

Relative syndecan-4/vinculin protein

Full length (FL)				Cellular fragment (CF)			
non-stimulated	Infliximab	TNFα	TNFα + Infliximab	non-stimulated	Infliximab	TNFα	TNFα + Infliximab
1,056	1,047	0,839	1,043	1,189	2,668	2,348	2,316
0,878	0,918	1,021	1,562	0,803	2,533	2,563	2,786
1,066	1,228	1,304	1,416	1,008	2,541	2,832	2,865
1,235	0,969	2,072	1,762	0,98	1,133	3,821	3,555
0,713	0,864	1,499	1,581	0,735	1,961	4,228	3,974
1,052	1,499	2,411	2,204	1,286	3,153	4,872	6,009
1,047	1,413	1,477	1,392		1,196	2,161	4,77
1,074	1,387	1,796	1,683	1,124	0,598	1,948	2,744
0,878	1,099	1,568	1,861	0,876	1,697	2,382	1,58
0,725	1,451	2,704	5,611	0,562	3,237	7,19	9,374
0,94	2,191	7,188	8,449	1,059	2,917	9,68	9,561
1,335	1,1	2,995	5,629	1,379	2,849	7,407	10,19
